# Deep-Ultraviolet Transparent Electrode Design for High-Performance and Self-Powered Perovskite Photodetector

**DOI:** 10.3390/nano13222979

**Published:** 2023-11-20

**Authors:** Thi My Huyen Nguyen, Manh Hoang Tran, Chung Wung Bark

**Affiliations:** Department of Electrical Engineering, Gachon University, 1342 Seongnam-daero, Sujeong-gu, Seongnam-si 13120, Gyeonggi-do, Republic of Korea; myhuyen.dho.k12@gmail.com (T.M.H.N.); tranmanhhoang1214@gmail.com (M.H.T.)

**Keywords:** halide perovskite photodetector, self-powered photodetector, 254 nm UVC detection, high responsivity, high on/off ratio

## Abstract

In this study, a highly crystalline and transparent indium-tin-oxide (ITO) thin film was prepared on a quartz substrate via RF sputtering to fabricate an efficient bottom-to-top illuminated electrode for an ultraviolet C (UVC) photodetector. Accordingly, the 26.6 nm thick ITO thin film, which was deposited using the sputtering method followed by post-annealing treatment, exhibited good transparency to deep-UV spectra (67% at a wavelength of 254 nm), along with high electrical conductivity (11.3 S/cm). Under 254 nm UVC illumination, the lead-halide-perovskite-based photodetector developed on the prepared ITO electrode in a vertical structure exhibited an excellent on/off ratio of 1.05 × 10^4^, a superb responsivity of 250.98 mA/W, and a high specific detectivity of 4.71 × 10^12^ Jones without external energy consumption. This study indicates that post-annealed ITO ultrathin films can be used as electrodes that satisfy both the electrical conductivity and deep-UV transparency requirements for high-performance bottom-illuminated optoelectronic devices, particularly for use in UVC photodetectors.

## 1. Introduction

Ultraviolet photodetectors (UV PDs) are optoelectronic devices that convert UV radiation into electrical signals. Recently, scholars have increasingly focused on UV PDs owing to their multiple applications, such as fire monitoring, partial discharge detection, image sensors, wireless communication, missile warning systems, and other indispensable applications in security surveillance [1,2,3,4,5]. It is well known that UV radiation is categorized into three bands depending on the wavelength: UVA (400–315 nm), UVB (315–280 nm), and UVC (280–100 nm) [6]. In comparison with relatively long-wavelength UVA, UVB and UVC have played crucial roles in the development of mankind [7]. For instance, Abajo et al. [7] proposed UVC light as a short-term and efficient strategy to limit the virus spread in the current SARS-CoV-2 pandemic. Moreover, in addition to food and supplements, the UVB spectrum is regarded as an essential source for vitamin D production to avoid the risks of diabetes, rickets, and bone metabolism [8].

Generally, UV PDs can be classified into two branches. The first one is photomultiplier tubes (PMTs), which enable multiplying the current signal induced by the incident photon by as much as 10^8^ times [9]. Although this takes advantage of the detectability of ultralow light intensity, the practical implementation of PMTs is hindered by the requirements of high energy consumption and high-vacuum-pressure operation, which are critical issues for portable applications and integrability into small-sized optoelectronic devices [10]. On the contrary, the second class—semiconductor-based PDs—can operate with self-powered or low-energy-consumption features in the ambient atmosphere, allowing various applications in practice. This kind of device can create a light-induced electrical signal via two stages: (i) semiconductors as photosensitive materials are excited to generate electron–hole pairs when exposed to light; (ii) the photogenerated electron–hole pairs can be effectively separated and driven toward the anode and cathode in the opposite direction. Typically, the bandgap and photoabsorption coefficient of semiconductors are responsible for the photon-to-charge conversion (i.e., stage (i)); meanwhile, the quality of the semiconductor (e.g., the charged carrier-trapping site, and interfacial and structural defects) and device architecture are responsible for the charge-to-signal conversion (i.e., stage (ii)) [6]. Focusing on photosensitive materials, many strategies of utilizing wide-bandgap semiconductors (e.g., Ga_2_O_3_, ZnO, TiO_2_, and SnO_2_) have been developed to adapt to the requirement of high UV selectivity, which allows PDs to be insensitive to the visible–infrared spectrum [11,12,13,14]. However, wide-bandgap material-based devices commonly stick to a low photoresponse because of the intrinsic low conductivity of photoactive components. To overcome this issue, a complicated architecture like a phototransistor is required. As a more straightforward approach, a device based on highly conductive narrow-bandgap semiconductors (e.g., Si) is equipped with an appropriate UV-pass filter to enhance the UV selectivity of the device. For example, X. Sheng et al. reported that a hybrid perovskite PD consisting of a narrow-bandgap Si as the photosensitive layer and a wide-bandgap Eu(DPEPO)(hfac)_3_ as the down-conversion filter exhibited both a high UV photoresponse and an acceptable UV selectivity [15]. Additional efforts have been made to detect broadband photodetection in the UV–NIR range employing a variety of photosensitive and high-mobility 2D materials, especially bandgap-tunable transition metal dichalcogenides like WS_2_ and ReSe_2_ [16,17]. In another view, organic–inorganic halide perovskite materials are promising semiconductors that can be comparable to conventional and expensive silicon [18]. These materials possess abundant precursors and simple production processes at low temperatures but can achieve high performance and facile large-scale production. Additionally, perovskite materials have high optical absorption coefficients, long charge carrier diffusion lengths, tunable bandgaps (1.4–2.3 eV), and low defect densities, which offer immense potential for high-sensitivity UV PDs [19,20,21,22,23].

Recently, several studies have reported that PDs based on perovskite materials exhibit broadband detection from near-infrared radiation to X-ray wavelengths [24,25]. In fact, to detect the deep-UV region (i.e., UVC and vacuum UV), it is necessary to consider the configuration of the PD and the direction of the incoming radiation. Considering PDs in a lateral structure, perovskite PDs usually require patterning procedures for the electrodes. However, the solubility of perovskite materials in water and polar solvents makes it difficult to design top electrodes using photolithography [26,27]. In addition, precise control of the electrode space is challenging because a wide electrode spacing requires a high external bias supply. To address this problem, perovskite materials are spin-coated onto pre-patterned electrode-coated substrates; however, the obtained perovskite film lacks uniformity [28]. Regarding PDs in a vertical structure, perovskite PDs in the bottom-up approach tend to utilize electrode materials that allow UV radiation to penetrate. Many candidates for transparent electrodes have been developed, such as transparent conductive oxides (TCOs) [29], transparent conductive polymers [30], graphene monolayers [31], nanofibers [32], metal nanowires [33], metal mesh [34,35], and ultrathin metal layers [36]. According to their individual characteristics, each of these electrode materials has an essential purpose. In general, electrical conductivity and optical transmittance at a short wavelength, which permits UV light to pass through, are the two factors that can be used to evaluate and compare the optoelectronic characteristics of electrodes for UV PDs. Considering vertical PDs with a bottom-illuminated structure, a poly(3,4-ethylenedioxythiophene):poly(styrene-sulfonate) (PEDOT:PSS) electrode is usually used for enabling deep-UVC light to penetrate through the PEDOT:PSS into the active layer. A PEDOT:PSS electrode is considered a low-cost conducting polymer that has a simple spin-coating process; however, the drawback is that it degrades under humidity and high temperatures [37,38]. Metal nanowires have been applied to deep-UV PDs due to their excellent electrical properties and synthesis scalability; however, they tend to break during the dispersion (e.g., sonication) and deposition processes [39]. Another strategy has also been implemented using ultrathin metal layers or metal mesh in PDs, where the transparency of the Ni (20 nm)/Au (10 nm) mesh was approximately 50% at a 350 nm wavelength [40]. Moreover, carbon-based transparent conducting electrodes (e.g., graphene and carbon nanotubes) have been utilized because of their remarkable thermal stability, high optical transparency, and good electrical conductivity. Nevertheless, their synthesis comprises several growth-and-transfer stages as well as chemical vapor deposition in a restricted environment, so this has yet to satisfy the requirements of industrial protocols [41]. To date, indium tin oxide (ITO) has been extensively used as a conventional electrode. For the majority of ITO electrodes, higher thickness can be equivalent to higher electrical conductivity at the expense of optical transparency, and vice versa. That is, a commercial ITO electrode with a thickness of ≥100 nm displays excellent electrical conductivity (sheet resistance~10–15 Ω/sq), but a restriction at a wavelength of 300 nm, which almost blocks the transmittance of UVC rays. Meanwhile, a high transparency ITO electrode exhibits a low conductivity. For example, M. Mazur and co-workers reported a remarkable decline in an ITO electrode’s conductivity from 0.17 S/cm to 1.69 × 10^−4^ S/cm when the thickness decreased from 270 nm to 30 nm [42]. Hence, the finding of the common trade-off between optical transmittance in short wavelengths and electrical conductivity has a significant impact on studying PDs in deep-UV detection.

In this study, we prepared ITO thin films with the magnetron sputtering method, which is the most widely employed approach for either laboratory-scale or industrial devices. This technology is based on the plasma excitation of a supplied gas (e.g., Ar or O_2_) that is ignited using a radio-frequency power source. A target material is bombarded with the plasma, which causes a transfer of particles from the constant target to the substrate. As a consequence, the prepared ITO film exhibits not only outstanding optoelectronic performance but also capacity repeatability for commercial production. The ITO transparency is implemented by regulating the thickness in the sputtering time step. Moreover, we utilized a post-annealing treatment for an ITO thin film to improve not only the crystallinity but also thin-film continuity. According to Naser M. Ahmed and co-workers, the grain size of ITO was found to increase with the annealing step, leading to the enhancement of the optical and electrical properties of the ITO thin films [43]. Thereby, we fabricated the ITO thin films with various sputtering times at room temperature to select the optimum transparency in the UVC region and applied a post-annealing treatment as an effective resolution for the conflict between the deep-UV transparency and the electrical conductivity. As a result, the prepared ITO electrode possesses good transparency to deep-UV spectra along with high electrical conductivity. In summary, this study presents an ITO thin film with a thickness of 26.6 nm as a promising electrode for a vertical perovskite PD under bottom-to-top illumination. Owing to a simple post-annealing treatment for a fast-deposition ITO thin film, the obtained ITO electrode displays exceptional optical transmission in the deep-UVC region (67% at 254 nm) and high electrical conductivity (11.3 S/cm) for charge transfer in PDs. As a proof-of-concept demonstration, the perovskite PD using the prepared ITO-coated quartz substrate exhibits outstanding performance at zero bias with an on/off ratio of >10^4^ and responsivity of 250 mA/W under 254 nm UVC illumination. Thus, this finding provides a simple but efficient pathway to prepare easily scalable transparent electrodes for futuristic optoelectronic devices demanding operation in the deep-UV region.

## 2. Experimental Details

### 2.1. Materials

Lead (II) iodide (PbI_2_, 99.999%), *N*,*N*-dimethylformamide (DMF, 99.8%), dimethyl sulfoxide (DMSO, 99.9%), 2,2′,7,7′-tetrakis[*N*,*N*-di(4-methoxyphenyl)amino]-9,9′-spirobifluorene (spiro-OMeTAD, 99%), isopropyl alcohol (IPA, 99.5%), chlorobenzene (99.8%), 4-tert-butyl pyridine (96%), lithium bis(trifluoromethane sulfonyl)imide (99.95%), and acetonitrile (99.93%) were purchased from Sigma-Aldrich (Seoul, Republic of Korea). Formamidinium iodide (FAI), methylammonium bromide (MABr), and methylammonium chloride (MACl) were procured from GreatCell Solar (Queanbeyan, Australia). The commercial ITO target (25.4 mm × 3 mm) was purchased from RND Korea company (Gwangmyeong, Republic of Korea).

### 2.2. Preparation of ITO Thin Films

First, 2 × 2 cm^2^ quartz substrates were cleaned in an ultrasonic bath for 10 min using acetone, 2-propanol, deionized water, and ethanol. Subsequently, ITO thin films were deposited on cleaned quartz substrates using a magnetron-sputtering instrument (Korea Vacuum Tech., Gimpo, Republic of Korea). The sputtering growth of the transparent conductive ITO films was implemented using a commercial ITO ceramic target with a diameter of 1 in. During the sputtering process, argon and oxygen were introduced at 30 and 1.2 SCCM, respectively. The working pressure was controlled at 50 mTorr, and the supply power was set to 50 W. The ITO thin films were prepared under different sputtering times (150, 300, 600, and 900 s) at room temperature, and then the obtained thin films were post-annealed at 450 °C for 1 h in a furnace for the crystallization of the ITO. The preparation of ITO thin films with the sputtering method is summarized in Table 1.

### 2.3. Preparation of UV Photodetector

The perovskite-based PDs were fabricated with a vertical structure and bottom-to-top illumination. Accordingly, a halide perovskite layer was deposited onto the obtained ITO specimens using a two-step spin-coating method, as described in our previous report [29]. That is, a PbI_2_ (1.3 M) solution (600 mg, dissolved in 950 µL of DMF and 50 µL of DMSO) was spin-coated onto the ITO layer at 2000 rpm for 30 s and annealed at 70 °C for 1 min. In the second step, an organic halogen mixed solution of FAI (60 mg), MABr (6 mg), and MACl (6 mg) in 1 mL of IPA was spin-coated on the PbI_2_ layer at 4000 rpm for 20 s (loading time of 20 s), followed by annealing at 150 °C for 15 min to form the perovskite layer. Subsequently, the hole transport layer (HTL) was deposited at 4000 rpm for 30 s by spin-coating the spiro-OMeTAD/chlorobenzene solution (80 mg/mL) with the addition of 4-tert-butylpyridine (28.8 μL) and 17.5 μL of lithium bis(trifluoromethanesulfonyl)imide/acetonitrile (52 mg/mL). Finally, Au (100 nm) was thermally evaporated onto the HTL to form the top electrode. The active area of the PD was determined to be 0.053 cm^2^ based on the overlap region of the ITO (bottom electrode) and a metal mask (Au top electrode).

### 2.4. Characterizations and Measurements

The crystallization of ITO thin films as-grown and post-annealing was characterized using an X-ray diffractometer (XRD; Rigaku DMAX 2200, Tokyo, Japan) with a Cu Kα radiation (λ = 1.506 Å) source at a scan rate of 2° min^−1^. The transmittances of the ITO-coated quartz substrates at different sputtering times and the absorbance of the full device were measured using an ultraviolet–visible spectrophotometer (UV–vis; Varian, Cary 50, San Diego, CA, USA). The sheet resistances of the prepared ITO thin films were measured using a four-point probe system (CMT-SR2000N, Suwon, Republic of Korea). The surface morphologies of the ITO thin film and the perovskite layer were analyzed using scanning electron microscopy (SEM; Hitachi, SU8600, Tokyo, Japan). Current–voltage (I-V) and current–time (I-t) plots were obtained using a Keithley 2410 source meter (SourceMeter, Cleveland, OH, USA) connected to a UV lamp (254 nm, Analytik, Jena, LA, USA). The light intensity was regulated via the distance between the UV lamp and the PD. It was determined using a commercial silicon photodiode (S120VC power head, Thorlabs, Tokyo, Japan). The response time of the PD was extracted from an individual I-t plot, which was measured using a mechanical shutter with an on/off state of light illumination. The EQE was measured using the solar McScience K3100 system (Suwon, Republic of Korea).

## 3. Results and Discussion

**Figure 1:**
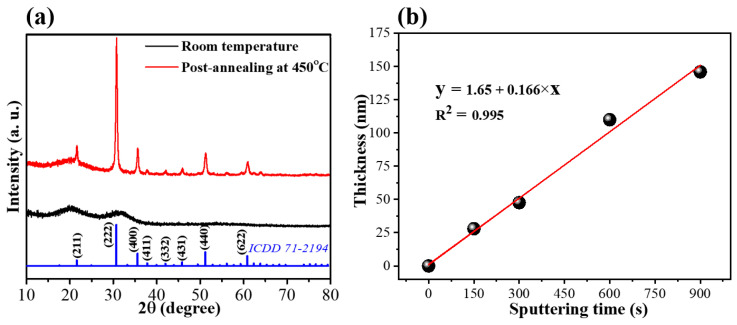
(**a**) XRD patterns of ITO thin films with/without post-annealing process. (**b**) Height profile of post-annealed ITO film with various sputtering times.

Figure 1a shows the XRD patterns of the ITO thin films sputtered onto quartz substrates with and without post-annealing in air. Unlike the amorphous phase of the ITO film sputtered at room temperature, the post-annealed ITO film exhibits diffraction peaks at 21.6, 30.7, 35.6, 37.9, 42.1, 45.9, 51.3, and 60.9° corresponding to the (211), (222), (400), (411), (332), (431), (440), and (622) planes of cubic In_2_O_3_ (ICDD 71-2194), respectively. This indicates that the post-annealing efficiently enhanced the crystallinity of the ITO thin films, which is consistent with the results of a previous study [43]. To investigate the effect of the electrode thickness on the deep-UV PD, the thickness of the post-annealed ITO thin film was controlled by varying the sputtering time. The height profile of the ITO thin film (Figure 1b) obtained from the FIB-SEM images (Figure S1) was linearly dependent on the deposition time. Accordingly, the thicknesses of the prepared ITO electrodes were 26.6, 51, 101, and 151 nm, corresponding to sputtering times of 150, 300, 600, and 900 s, respectively.

**Figure 2:**
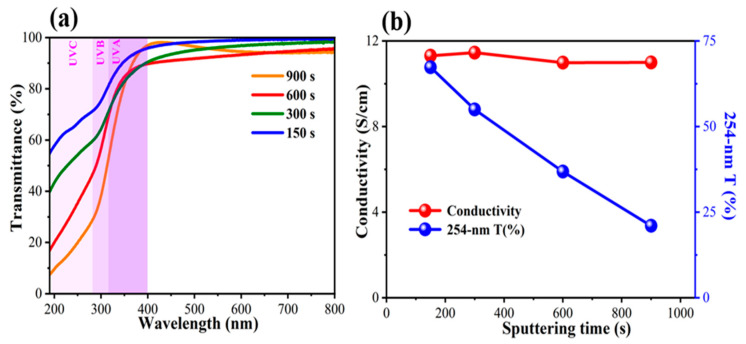
(**a**) UV–vis spectra; (**b**) 254 nm transmittances and electrical conductivities of ITO thin films with different deposition times.

Figure 2a shows the UV–vis spectra of the ITO thin films at different deposition times. It is reasonable to assume that the decrease in ITO thickness resulted in increased transmittance in the UV spectrum, especially in the UVB-C region. Considering the wavelength of 254 nm light, the transmittance of ITO can reach 67% with a deposition time as short as 150 s (Figure 2b). Interestingly, the conductivity of the ITO thin film is independent of the thickness of the conductivity of the 26.6 nm thick ITO thin film to as high as 11.3 S/cm (Figure 2b). As mentioned in previous studies, the conductivity of ITO electrodes prepared via sputtering without post-annealing treatment tends to decrease as the thickness of the thin film decreases. For instance, M. Mazur reported a remarkable decline in ITO electrode conductivity from 0.17 S/cm (at 270 nm) to 1.69 × 10^−4^ S/cm (at 30 nm) [42]. This indicates that a simple post-annealing treatment plays a crucial role in improving the electrical conductivity of an ITO electrode. This is because the post-annealing process can increase the ITO grain size and alleviate the negative effect on charge transfer originating from nanosized voids and grain boundaries [43]. Although the decrease in thickness sacrificed the low sheet resistance (Figure S2), which is favorable for the horizontal charge transfer in a lateral structure, this work suggests that 26.6 nm thick ITO thin-film-coated quartz is a promising electrode for vertical-structured UVC PDs, which require both high electrical conductivity for carrier collection and high transparency to UVC light for efficient penetration of the active layer (herein referred to as the halide perovskite layer).

**Figure 3:**
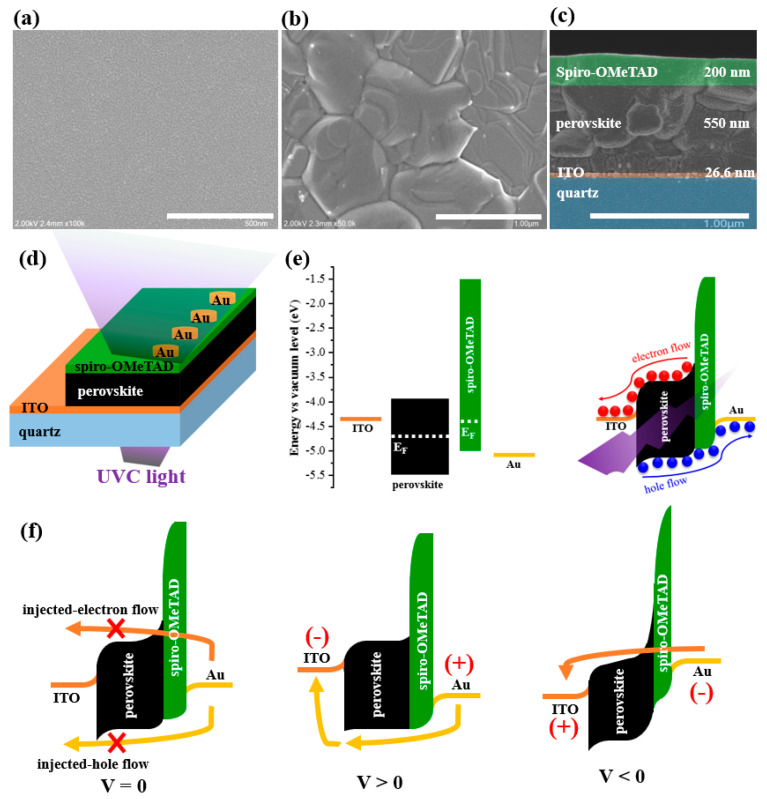
SEM images of (**a**) the 26.6 nm thick ITO thin film and (**b**) halide perovskite film prepared on the ITO thin film. (**c**) Cross-sectional SEM image of quartz/ITO/perovskite/spiro-OMeTAD block. (**d**) Schematic architecture and operating mechanisms of UVC detector (**e**) under light conditions and (**f**) under dark conditions.

Figure 3a,b shows the surface SEM images of a 26.6 nm thick ITO electrode and a halide perovskite film prepared using the solution process onto the ITO layer. The ITO thin film appears to be continuous and smooth without cracks or cavities, facilitating the growth of a large-grained and compact perovskite active layer with a thickness of 550 nm (Figure 3c). To fabricate the self-powered UVC PD, a p-type 200 nm thick spiro-OMeTAD polymer and a Au film were applied to the perovskite layer as the hole-transfer layer and hole-collecting electrode, respectively, as shown in Figure 3d [29]. The operating mechanism is shown in Figure 3e [44,45,46]. Accordingly, after the constituent layers make contact, their Fermi levels are aligned to form an ohmic contact at the ITO/perovskite interface and a p-n junction at the perovskite/spiro-OMeTAD interface. Upon UVC irradiation, owing to the highly transparent conductive electrode, UV light can fully approach the active perovskite region, allowing numerous photoexcited electron–hole pairs. On the one hand, the ohmic ITO/perovskite contact facilitates the movement of electrons from the perovskite to the ITO electrode. On the other hand, the p-n perovskite/spiro-OMeTAD junction not only hinders the transfer of electrons toward the Au side but also efficiently injects holes toward the Au electrode. As a result, the flow of photogenerated electrons and holes can be easily swept toward the ITO and Au electrodes, respectively, without energy consumption. In addition, to evaluate the effect of ITO thickness on the photodetector performance, devices were constructed in the structure, as shown in Figure 3d, using different ITO thicknesses (with deposition times of 150, 300, and 600 s). As expected, the device with an ITO deposition time of 150 s (corresponding to 26.6 nm) exhibited the highest photoresponse to UVC light at 0 V (Figure S3). This is reasonable because the 26.6 nm thick ITO electrode satisfies the requirements of high transparency in the deep-UV spectrum and high electrical conductivity. Additionally, the photoresponse of the optimum device tended to increase with the detectable UV wavelength (Figure S4). In particular, the current-in-light of the device was 1.25, 2.4, and 3.2 µA as exposed to 254 nm UVC, 305 nm UVB, and 365-nm UVA at the same light intensity of ~107 µW/cm^2^, respectively, which coincides with the UV transmittance of the ITO electrode (67% at 254 nm, 75% at 305 nm, and 93% at 365 nm in Figure 2a). The plot of external quantum efficiency (EQE) as a function of wavelength (in the range of 300–800 nm; Figure S5a) showed a broadband detection in the UV-to-visible light region, consistent with the photoabsorption nature of the narrow-bandgap perovskite (Figure S5b). Moreover, the notable enhancement of the EQE in the UV region (from 15.8% to 54.7% at a wavelength of 300 nm) with the decrease in ITO thickness (from 101 nm to 26.6 nm) reveals that controlling the transparent electrode thickness played a vital role in improving the performance of the deep-UV PDs.

**Figure 4:**
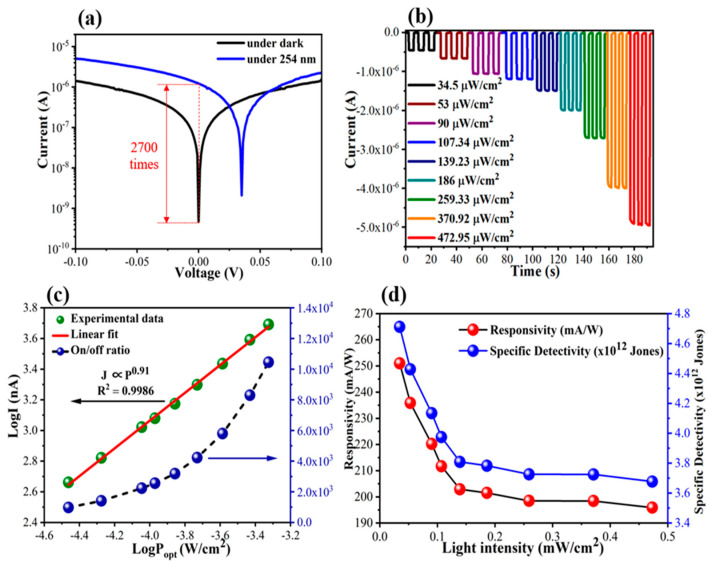
(**a**) I–V plots in darkness and under 254 nm UV illumination (107.34 µW/cm^2^). (**b**) I-t plots under different light intensities, (**c**) on/off ratio and the logarithm of photocurrent as a function of power intensity, and (**d**) responsivity and detectivity at 0 V under 254 nm UV illumination in the power density range of 34.5–472.95 µW/cm^2^.

Figure 4a shows semi-logarithmic I-V plots of the fabricated PD in darkness and under 254 nm UVC illumination at a low power density of 107.34 µW/cm^2^. At 0 V, the current-in-light (I_L_) is 2700 times higher than the dark current (I_D_), demonstrating that the device can operate efficiently under self-powered conditions. At an applied external voltage (regardless of positive or negative voltage), the I-V plot under dark conditions showed a significant increase in dark current, resulting in a strong reduction in the on/off ratio (only 1.4 and 3.6 at 0.1 and −0.1 V, respectively). The influence of external voltage on dark current is illustrated in Figure 3f. Briefly, without any bias (V = 0 V), the injected-electron flow and injected-hole flow are effectively hindered by intrinsic energy barriers formed at the perovskite/spiro-OMeTAD interface and the ITO/perovskite interface, respectively. However, under the positive bias (V > 0 V), the ITO/perovskite contact barrier is reduced and allows more holes injected from Au to the ITO electrode. Meanwhile, under the negative bias (V < 0 V), the perovskite/spiro-OMeTAD contact barrier becomes narrow (due to the Zener effect [47]) and allows electrons injected from Au to the ITO electrode. Figure 4b shows the I-t plots of the device at 0 V under 254 nm light at a power intensity (*P_opt_*) in the range of 34.5–472.95 µW/cm^2^. When the *P_opt_* increased, the I_L_ dramatically increased because the number of photogenerated electron–hole pairs throughout the perovskite layer increased. Consequently, the on/off ratio (defined as the I_L_/I_D_ ratio) of the device increased from 9.8 × 10^2^ (at 34.5 µW/cm^2^) to 1.05 × 10^4^ (at 472.95 µW/cm^2^), as shown in Figure 4c. Additionally, the photocurrent (I_P_) (defined as I_L_-I_D_) was well-fitted with a simple power function: $I_{P}\propto P_{opt}^{\gamma }$, where γ=0.91. The non-unity γ was an inevitable result of the carrier trapping and recombination process originating from the boundaries between the perovskite grains and the surface defects [48,49,50]. Thus, the responsivity (R) and specific detectivity (D*) calculated using Equations (1) and (2) tended to decrease with *P_opt_* in with the following relationship: $R\propto P_{opt}^{\gamma -1}$, where γ=0.91 (Figure 4d).

(1) $$R=\frac{I_{P}}{P_{opt}S}$$

(2) $$D^{*}=\frac{RS^{0.5}}{{\left(2eI_{D}\right)}^{0.5}}$$

where S denotes the active area of the device, e denotes the electron charge, h denotes Planck’s constant, and c indicates the speed of light.

In addition to photocurrent, which can be obtained in short-circuit mode, another important photoresponse signal governing self-powered PDs is photovoltage (V_P_), which is measured in open-circuit mode. Similar to the I_P_, the V_P_, defined as the different oper-circuit voltage (V_OC_) of the device under dark and light conditions, gradually increases with the increase in the *P_opt_*. Figure S6a shows V-t plots of the ITO/perovskite/spiro-OMeTAD/Au device under 254 nm UV illumination at different light intensities. As exhibited in Figure S6b, while the V_OC_-in-dark remained unchanged at the level of 10^−4^ V, the V_OC-_in-light was at the level of 10^−1^ V with a tendency to increase with light intensity. The generation of V_P_ is a consequence of three consecutive stages. Firstly, (i) the Fermi levels of constituent layers (including perovskite and spiro-OMeTAD) are aligned to keep the negligible difference in the voltages between two electrodes. Then, (ii) upon UV light illumination, the photogenerated electrons and holes in the photosensitive perovskite are self-driven toward the ITO and spiro-OMeTAD sides in the opposite direction owing to the favorable energy band diagram (as mentioned in Figure 3e). Finally, (iii) when the net current is retained at zero due to the open-circuit mode, the photogenerated electrons and holes are accumulated at the ITO and spiro-OMeTAD sides, resulting in the splitting of the Fermi levels.

**Figure 5:**
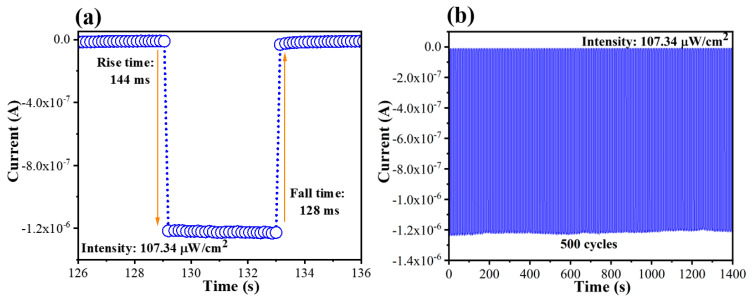
(**a**) Individual I-t plot and (**b**) multi-cycle I-t plot of the device at 0 V under 254 nm UV illumination (107.34 µW/cm^2^).

The response time of the device was calculated based on an individual on/off cycle. Accordingly, the rise/fall time was estimated as the periods in which the photocurrent increased from 10% to 90% and dropped from 90% to 10% of its maximum level, respectively. As shown in Figure 5a, the response speed of the device was determined to be 144 ms (rise time) and 128 ms (fall time). Furthermore, the I-t plot continuously recording 500 on/off cycles displayed a durable UVC photoresponse of the device (Figure 5b), suggesting the good stability of the device for practical applications. In comparison with recent perovskite-based PDs and some inorganic/organic-semiconductor-based PDs (e.g., SiC, Alq3, and Eu-MOF), our device exhibited a superior on/off ratio, R, and D* values, even when operated at zero bias (as shown in Table 2). In particular, owing to the success of building a highly UVC transparent and highly conductive ITO electrode, this work advocated for R (250.98 mA/W) and D* (4.71 × 10^12^ Jones) values that were significantly higher than the 52.7 mA/W and 4.65 × 10^11^ Jones of the device in our previous report, which had a similar structure but used a 150 nm thick ITO electrode [29]. In summary, this work proposed a strategy that is a combination of (i) the fast deposition of ITO to obtain an ultrathin electrode, and (ii) the implementation of a simple post-annealing treatment to enhance ITO crystallinity. While factor (i) allowed a high UV transparency with a 254 nm transmittance of 67%, factor (ii) allowed a high electrical conductivity of 11.3 S/cm. Hence, this finding demonstrated a facile and effective process to achieve an efficient electrode for optoelectronics, especially deep-UV photodetectors.

## 4. Conclusions

We successfully prepared a thin film of a 26.6 nm thick ITO electrode on a quartz substrate that exhibited good transmittance in the UVC region (67% at 254 nm) and high electrical conductivity owing to a post-annealing treatment of a fast-deposition ITO thin film. Therefore, the halide-perovskite-based UV PD can operate under bottom-to-top illuminated 254 nm UVC light. The fabricated device exhibited excellent performance in the self-powered mode with a remarkable responsivity of 250.98 mA/W, a high specific detectivity of 4.71 × 10^12^ Jones, and an outstanding on/off photocurrent ratio (1.05 × 10^4^). This study demonstrated an efficient ITO-coated quartz substrate for the preparation of bottom-illuminated optoelectronic devices. In addition to well-known top-electrodes like graphene mono-/few-layer and silver nanowires, a thermally stable and highly transparent bottom-electrode like the prepared ITO thin film is believed to accelerate the development of see-through electronics in the future.

## Figures and Tables

**Table 1:** Fabrication parameters of ITO thin films.

Parameter	Value
Target material	ITO (90 wt.% In_2_O_3_: 10 wt.% SnO_2_)
Substrate	Quartz
Working pressure (mTorr)	50
Sputtering power (W)	50
Gas flow rate (SCCM)	Ar:O_2_ = 30:1.2
Sputtering time (second)	150, 300, 600, and 900
Substrate temperature	Room temperature
Post-annealed temperature/time	450 °C/1 h

**Table 2:** Performance comparison between the prepared perovskite photodetector and recent narrow-bandgap semiconductor-based deep UV photodetectors.

Device Structure	Light (nm)	Bias (V)	Responsivity (mA/W)	Detectivity (Jones)	On/Off Ratio	Ref./Year
ITO/perovskite/spiro-OMeTAD/Au	254	0	250.98	4.71 × 10^12^	1.05 × 10^4^	This study
PH1000/SnO_2_/perovskite/spiro-OMeTAD/Au	254	0	5.64	4.03 × 10^11^		[51]/2023
ITO/SnO_2_/perovskite/spiro-OMeTAD/Au	254	2	50.8	4.47 × 10^13^		[52]/2022
PH1000/perovskite/spiro-OMeTAD/PEDOT: PSS	254	0	7.16	5.2 × 10^11^	9.57 × 10^3^	[53]/2022
ITO/perovskite/ITO	254	−2	5.07	5.49 × 10^11^		[54]/2022
PEDOT: PSS/SnO_2_/perovskite/spiro-OMeTAD/Au	254	0	4.92	7.57 × 10^10^	337.14	[55]/2021
ITO/perovskite/spiro-OMeTAD/Au	254	0	52.68	4.65 × 10^11^	10^3^	[29]/2021
Si/SiO_2_/perovskite/Au	265	3	22.1	1.2 × 10^11^	22	[56]/2020
Perovskite/FTO/TiO_2_/perovskite/spiro-OMeTAD/Ag	254	0	49.4	1.2 × 10^12^		[57]/2018
Si/Au/SiC/Pt	250	10	180		68.75	[58]/2016
Cr/Au/4H-SiC/graphene/SiO_2_/Cr/Au	285	0	90	2.95 × 10^12^		[59]/2023
ITO/PEI/Eu-MOF/PEDOT:PSS/Au	254	0	0.24	1.015 × 10^10^	107.33	[30]/2022
ITO/PEDOT:PSS/TPD:Alq3/Al	365	0	0.522	3.11 × 10^10^		[60]/2021
ITO/PEDOT:PSS/NPD:Alq3/LiF/Al	365	0	1.07	1.04 × 10^11^	1.3 × 10^5^	[61]/2021

## Data Availability

Data are contained within the article.

## Supplementary Materials

The following supporting information can be downloaded at https://www.mdpi.com/article/10.3390/nano13222979/s1. Figure S1: Cross-sectional images of ITO thin films with sputtering times of 900, 600, 300, and 150 s; Figure S2: The sheet resistance of ITO thin films with different deposition times; Figure S3: I-t plots of the prepared photodetectors using different ITO thicknesses (deposition times of 150, 300, and 600 s) under 254 nm UV illumination (107.34 µW/cm^2^); Figure S4: The I-t plots of the prepared photodetector using 26.6 nm thick ITO thin film under 365 nm UVA, 302 nm UVB, and 254 nm UVC illumination (107.34 µW/cm^2^). Figure S5: (a) The external quantum efficiency (EQE) plot as a function of wavelength, and (b) UV–vis spectrum of the complete device; Figure S6: (a) The V-t plots in open-circuit mode of the prepared photodetector using 26.6 nm thick ITO thin film under 254 nm UV illumination, and (b) photovoltage as a function of light intensity.

## References

[1] Zeng L.H., Chen Q.M., Zhang Z.X., Wu D., Yuan H., Li Y.Y., Qarony W., Lau S.P., Luo L.B., Tsang Y.H. (2019). Multilayered PdSe_2_/perovskite Schottky junction for fast, self-powered, polarization-sensitive, broadband photodetectors, and image sensor application. Adv. Sci..

[2] Xia Y., Zhai G., Zheng Z., Lian L., Liu H., Zhang D., Gao J., Zhai T., Zhang J. (2018). Solution-processed solar-blind deep ultraviolet photodetectors based on strongly quantum confined ZnS quantum dots. J. Mater. Chem. C.

[3] Ding M., Zhao D., Yao B., Li Z., Xu X. (2015). Ultraviolet photodetector based on heterojunction of n-ZnO microwire/p-GaN film. RSC Adv..

[4] Zhang Z., Zheng W., Lin R., Huang F. (2018). High-sensitive and fast response to 255 nm deep-UV light of CH_3_NH_3_PbX_3_ (X = Cl, Br, I) bulk crystals. R. Soc. Open Sci..

[5] Kim H., Han J.S., Choi J., Kim S.Y., Jang H.W. (2018). Halide perovskites for applications beyond photovoltaics. Small Methods.

[6] Zou Y., Zhang Y., Hu Y., Gu H. (2018). Ultraviolet detectors based on wide bandgap semiconductor nanowire: A review. Sensors.

[7] Garcia de Abajo F.J., Hernández R.J., Kaminer I., Meyerhans A., Rosell-Llompart J., Sanchez-Elsner T. (2020). Back to normal: An old physics route to reduce SARS-CoV-2 transmission in indoor spaces. ACS Nano.

[8] Holick M.F. (2008). Sunlight, UV-radiation, vitamin D and skin cancer: How much sunlight do we need?. Sunlight, Vitamin D and Skin Cancer.

[9] Ball D.W. (2006). Field Guide to Spectroscopy.

[10] Xie C., Lu X.T., Tong X.W., Zhang Z.X., Liang F.X., Liang L., Luo L.B., Wu Y.C. (2019). Recent progress in solar-blind deep-ultraviolet photodetectors based on inorganic ultrawide bandgap semiconductors. Adv. Funct. Mater..

[11] Soci C., Zhang A., Xiang B., Dayeh S.A., Aplin D., Park J., Bao X., Lo Y.-H., Wang D. (2007). ZnO nanowire UV photodetectors with high internal gain. Nano Lett..

[12] Zhang D.-Y., Ge C.-W., Wang J.-Z., Zhang T.-F., Wu Y.-C., Liang F.-X. (2016). Single-layer graphene-TiO_2_ nanotubes array heterojunction for ultraviolet photodetector application. Appl. Surf. Sci..

[13] Hu L., Yan J., Liao M., Wu L., Fang X. (2011). Ultrahigh external quantum efficiency from thin SnO_2_ nanowire ultraviolet photodetectors. Small.

[14] Du J., Xing J., Ge C., Liu H., Liu P., Hao H., Dong J., Zheng Z., Gao H. (2016). Highly sensitive and ultrafast deep UV photodetector based on a β-Ga_2_O_3_ nanowire network grown by CVD. J. Phys. D Appl. Phys..

[15] Sheng X., Yu C., Malyarchuk V., Lee Y.H., Kim S., Kim T., Shen L., Horng C., Lutz J., Giebink N.C. (2014). Silicon-Based Visible-Blind Ultraviolet Detection and Imaging Using Down-Shifting Luminophores. Adv. Opt. Mater..

[16] Khan M.F., Ahmed F., Rehman S., Akhtar I., Rehman M.A., Shinde P.A., Khan K., Kim D.-K., Eom J., Lipsanen H. (2020). High performance complementary WS_2_ devices with hybrid Gr/Ni contacts. Nanoscale.

[17] Elahi E., Khan M.F., Rehman S., Khalil H.W., Rehman M.A., Kim D.-K., Kim H., Khan K., Shahzad M., Iqbal M.W. (2020). Enhanced electrical and broad spectral (UV-Vis-NIR) photodetection in a Gr/ReSe_2_/Gr heterojunction. Dalton Trans..

[18] Liu K., Jiang Y., Jiang Y., Guo Y., Liu Y., Nakamura E. (2018). Chemical formation and multiple applications of organic–inorganic hybrid perovskite materials. J. Am. Chem. Soc..

[19] Wang W., Xu H., Cai J., Zhu J., Ni C., Hong F., Fang Z., Xu F., Cui S., Xu R. (2016). Visible blind ultraviolet photodetector based on CH_3_NH_3_PbCl_3_ thin film. Opt. Express.

[20] Ka I., Gerlein L.F., Asuo I.M., Nechache R., Cloutier S.G. (2018). An ultra-broadband perovskite-PbS quantum dot sensitized carbon nanotube photodetector. Nanoscale.

[21] Lin Q., Armin A., Nagiri R.C.R., Burn P.L., Meredith P. (2015). Electro-optics of perovskite solar cells. Nat. Photonics.

[22] Ding J., Fang H., Lian Z., Lv Q., Sun J.-L., Yan Q. (2018). High-performance stretchable photodetector based on CH_3_NH_3_PbI_3_ microwires and graphene. Nanoscale.

[23] Asuo I.M., Fourmont P., Ka I., Gedamu D., Bouzidi S., Pignolet A., Nechache R., Cloutier S.G. (2019). Highly efficient and ultrasensitive large-area flexible photodetector based on perovskite nanowires. Small.

[24] Perumal Veeramalai C., Yang S., Zhi R., Sulaman M., Saleem M.I., Cui Y., Tang Y., Jiang Y., Tang L., Zou B. (2020). Solution-Processed, Self-Powered Broadband CH_3_NH_3_PbI_3_ Photodetectors Driven by Asymmetric Electrodes. Adv. Opt. Mater..

[25] Yakunin S., Sytnyk M., Kriegner D., Shrestha S., Richter M., Matt G.J., Azimi H., Brabec C.J., Stangl J., Kovalenko M.V. (2015). Detection of X-ray photons by solution-processed lead halide perovskites. Nat. Photonics.

[26] Wang H., Haroldson R., Balachandran B., Zakhidov A., Sohal S., Chan J.Y., Zakhidov A., Hu W. (2016). Nanoimprinted perovskite nanograting photodetector with improved efficiency. ACS Nano.

[27] Boyd C.C., Cheacharoen R., Leijtens T., McGehee M.D. (2018). Understanding degradation mechanisms and improving stability of perovskite photovoltaics. Chem. Rev..

[28] Lin D., Liu J., Haroldson R., Moon J., Li Z., Zakhidov A., Hu W., Gu Q. (2022). High-Performance Directly Patterned Nanograting Perovskite Photodetector with Interdigitated Electrodes. Adv. Opt. Mater..

[29] Nguyen T.M.H., Kim S., Bark C.W. (2021). Solution-processed and self-powered photodetector in vertical architecture using mixed-halide perovskite for highly sensitive UVC detection. J. Mater. Chem. A.

[30] Nguyen T.M.H., Bark C.W. (2022). Self-Powered UVC Photodetector Based on Europium Metal–Organic Framework for Facile Monitoring Invisible Fire. ACS Appl. Mater. Interfaces.

[31] Li Y., Zhou Z., Pan H., Chen J., Wang Y., Qu Q., Zhang D., Li M., Lu Y., He Y. (2023). High-performance Ga_2_O_3_/FTO-based self-driven solar-blind UV photodetector with thickness-optimized graphene top electrode. J. Mater. Res. Technol..

[32] Zhang H., Zhu X., Tai Y., Zhou J., Li H., Li Z., Wang R., Zhang J., Zhang Y., Ge W. (2023). Recent advances in nanofiber-based flexible transparent electrodes. Int. J. Extreme Manuf..

[33] Wang S., Wu C., Wu F., Zhang F., Liu A., Zhao N., Guo D. (2021). Flexible, transparent and self-powered deep ultraviolet photodetector based on Ag NWs/amorphous gallium oxide Schottky junction for wearable devices. Sens. Actuator A Phys..

[34] Zhu X., Liu M., Qi X., Li H., Zhang Y.F., Li Z., Peng Z., Yang J., Qian L., Xu Q. (2021). Templateless, plating-free fabrication of flexible transparent electrodes with embedded silver mesh by electric-field-driven microscale 3D printing and hybrid hot embossing. Adv. Mater..

[35] Li Z., Li H., Zhu X., Peng Z., Zhang G., Yang J., Wang F., Zhang Y.F., Sun L., Wang R. (2022). Directly printed embedded metal mesh for flexible transparent electrode via liquid substrate electric-field-driven jet. Adv. Sci..

[36] Guo D., Su Y., Shi H., Li P., Zhao N., Ye J., Wang S., Liu A., Chen Z., Li C. (2018). Self-powered ultraviolet photodetector with superhigh photoresponsivity (3.05 A/W) based on the GaN/Sn: Ga_2_O_3_ pn junction. ACS Nano.

[37] Vitoratos E., Sakkopoulos S., Dalas E., Paliatsas N., Karageorgopoulos D., Petraki F., Kennou S., Choulis S.A. (2009). Thermal degradation mechanisms of PEDOT: PSS. Org. Electron..

[38] Nardes A.M., Kemerink M., De Kok M., Vinken E., Maturova K., Janssen R. (2008). Conductivity, work function, and environmental stability of PEDOT: PSS thin films treated with sorbitol. Org. Electron..

[39] Cao W., Li J., Chen H., Xue J. (2014). Transparent electrodes for organic optoelectronic devices: A review. J. Photonics Energy.

[40] Chen D., Fan G., Zhang H., Zhou L., Zhu W., Xi H., Dong H., Pang S., He X., Lin Z. (2019). Efficient Ni/Au mesh transparent electrodes for ITO-free planar perovskite solar cells. Nanomaterials.

[41] Liu W.-W., Chai S.-P., Mohamed A.R., Hashim U. (2014). Synthesis and characterization of graphene and carbon nanotubes: A review on the past and recent developments. J. Ind. Eng. Chem..

[42] Mazur M., Pastuszek R., Wojcieszak D., Kaczmarek D., Domaradzki J., Obstarczyk A., Lubanska A. (2022). Effect of thickness on optoelectronic properties of ITO thin films. Circuit World.

[43] Ahmed N.M., Sabah F.A., Abdulgafour H., Alsadig A., Sulieman A., Alkhoaryef M. (2019). The effect of post annealing temperature on grain size of indium-tin-oxide for optical and electrical properties improvement. Results Phys..

[44] Schulz P., Tiepelt J.O., Christians J.A., Levine I., Edri E., Sanehira E.M., Hodes G., Cahen D., Kahn A. (2016). High-work-function molybdenum oxide hole extraction contacts in hybrid organic–inorganic perovskite solar cells. ACS Appl. Mater. Interfaces.

[45] Seok H.-J., Ali A., Seo J.H., Lee H.H., Jung N.-E., Yi Y., Kim H.-K. (2019). Zno: Ga-graded ITO electrodes to control interface between PCBM and ITO in planar perovskite solar cells. Sci. Technol. Adv. Mater..

[46] Tonui P., Oseni S.O., Sharma G., Yan Q., Mola G.T. (2018). Perovskites photovoltaic solar cells: An overview of current status. Renew. Sustain. Energy Rev..

[47] Tran M.H., Nguyen T.M.H., Bark C.W. (2023). Facile fabrication of low-defect spinel zinc ferrite oxide thin film for high-performance ultraviolet photodetector. J. Alloys Compd..

[48] Zhang Z.-X., Li C., Lu Y., Tong X.-W., Liang F.-X., Zhao X.-Y., Wu D., Xie C., Luo L.-B. (2019). Sensitive deep ultraviolet photodetector and image sensor composed of inorganic lead-free Cs_3_Cu_2_I_5_ perovskite with wide bandgap. J. Phys. Chem. Lett..

[49] Fang H., Hu W. (2017). Photogating in low dimensional photodetectors. Adv. Sci..

[50] Zhang T., Wang F., Zhang P., Wang Y., Chen H., Li J., Wu J., Chen L., Chen Z.D., Li S. (2019). Low-temperature processed inorganic perovskites for flexible detectors with a broadband photoresponse. Nanoscale.

[51] Choi G.I., Bark C.W., Choi H.W. (2023). Study on a Mixed-Cation Halide Perovskite-Based Deep-Ultraviolet Photodetector. Coatings.

[52] Hong S.B., Choi H.W. (2022). A Study on UVC Photodetector Using Mixed-Cation Perovskite with High Detection Rate as Light-Absorption Layer. Nanomaterials.

[53] Nguyen T.M.H., Garner S.M., Bark C.W. (2022). Metal Electrode-Free Halide Perovskite-Based Flexible Ultraviolet-C Photodetector with Large Area. Nanoscale Res. Lett..

[54] Choi G.I., Choi H.W. (2022). A study to improve the performance of mixed cation–halide perovskite-based UVC photodetectors. Nanomaterials.

[55] Nguyen T.M.H., Lee S.K., Kim S., Bark C.W. (2021). Practical demonstration of deep-ultraviolet detection with wearable and self-powered halide perovskite-based photodetector. ACS Appl. Mater. Interfaces.

[56] Yang J., Kang W., Liu Z., Pi M., Luo L.-B., Li C., Lin H., Luo Z., Du J., Zhou M. (2020). High-performance deep ultraviolet photodetector based on a one-dimensional lead-free halide perovskite CsCu_2_I_3_ film with high stability. J. Phys. Chem. Lett..

[57] Tong G., Li H., Zhu Z., Zhang Y., Yu L., Xu J., Jiang Y. (2018). Enhancing hybrid perovskite detectability in the deep ultraviolet region with down-conversion dual-phase (CsPbBr_3_–Cs_4_PbBr_6_) films. J. Phys. Chem. Lett..

[58] Aldalbahi A., Li E., Rivera M., Velazquez R., Altalhi T., Peng X., Feng P.X. (2016). A new approach for fabrications of SiC based photodetectors. Sci. Rep..

[59] Jehad A.K., Fidan M., Ünverdi Ö., Çelebi C. (2023). CVD graphene/SiC UV photodetector with enhanced spectral responsivity and response speed. Sens. Actuator A Phys..

[60] Basir A., Alzahrani H., Sulaiman K., Muhammadsharif F.F., Abdullah S.M., Mahmoud A.Y., Bahabry R.R., Alsoufi M.S., Bawazeer T.M., Ab Sani S.F. (2021). A novel self-powered photodiode based on solution-processed organic TPD: Alq3 active layer. Mater. Sci. Semicond. Process..

[61] Alzahrani H., Sulaiman K., Mahmoud A.Y., Bahabry R.R. (2021). Ultrasensitive self-powered UV photodetector based on a novel pn heterojunction of solution-processable organic semiconductors. Synth. Met..

