# Supplementary material for: Deep-Ultraviolet Transparent Electrode Design for High-Performance and Self-Powered Perovskite Photodetector
Source: Nanomaterials (Basel). 2023 Nov 20;13(22):2979. doi: 10.3390/nano13222979 (PMC10675135; doi:10.3390/nano13222979)
Supplement: Supplementary file 1 [file nanomaterials-13-02979-s001.zip › nanomaterials-2708657-supplementary.pdf]

Supplementary Information

Deep-Ultraviolet Transparent Electrode Design for High-Performance and Self-Powered Perovskite Photodetector

Thi My Huyen Nguyen, Manh Hoang Tran and Chung Wung Bark *

Department of Electrical Engineering, Gachon University, 1342 Seongnam-daero, Sujeong-gu, Seongnam-si 13120, Gyeonggi-do, Korea; myhuyen.dho.k12@gmail.com (T.M.H.N.); tranmanhhoang1214@gmail.com (M.H.T.)

* Correspondence: bark@gachon.ac.kr; Tel.: +82-31-750-5351

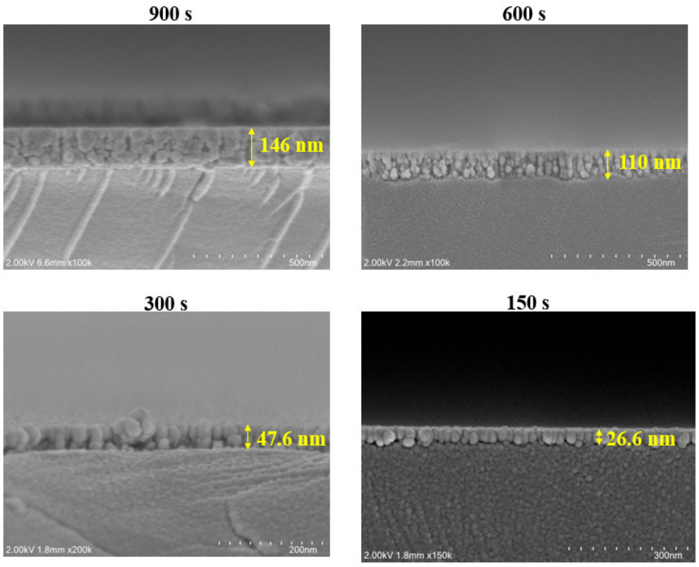

Figure S1. Cross-section images of ITO thin films at sputtering times of 900, 600, 300, and 150 s.

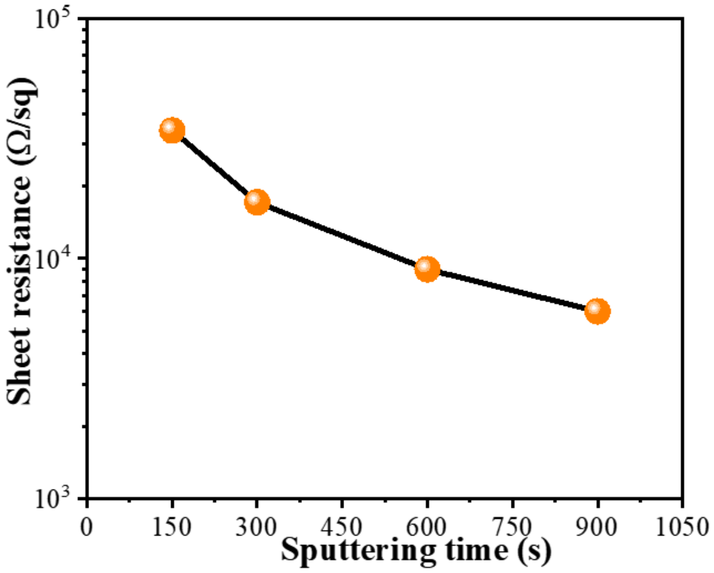

Figure S2. The sheet resistance of ITO thin films with different deposition times.

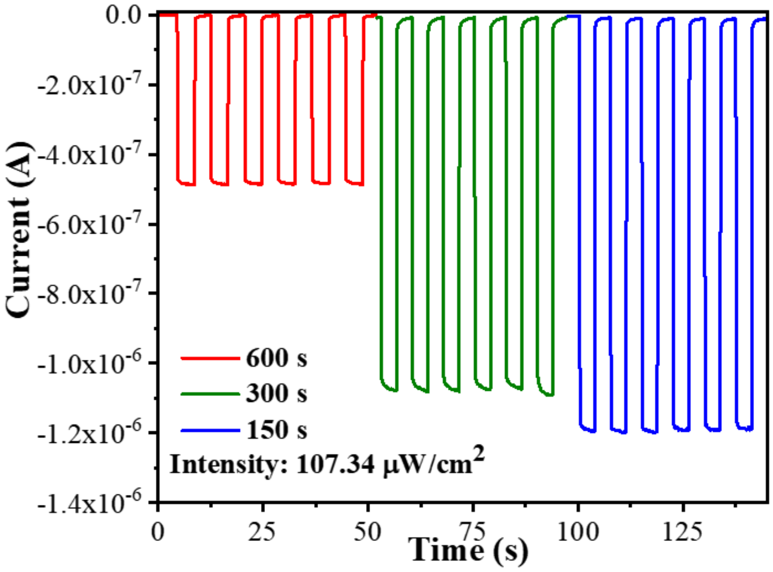

Figure S3. I-t plots of the prepared photodetectors using different ITO thicknesses (deposition times of 150, 300, and 600 s) under 254-nm UV illumination (107.34 μW/cm²).

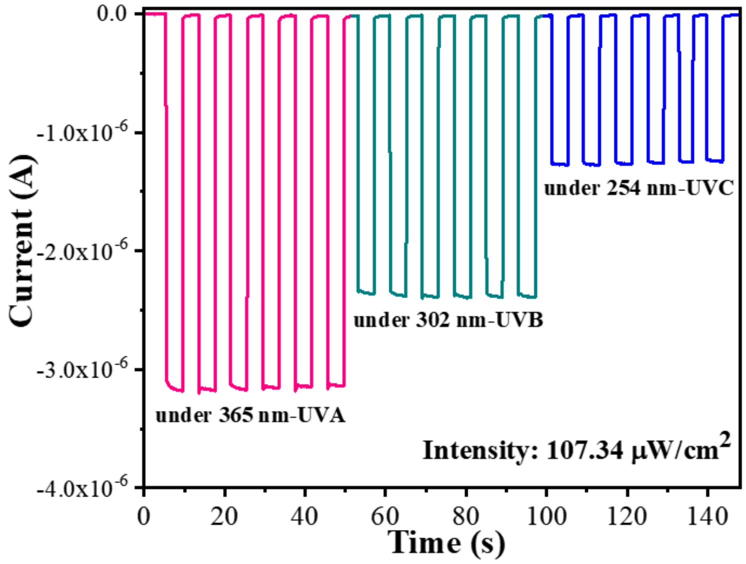

Figure S4. The I-t plots of the prepared photodetector using 26.6 nm-thick ITO thin film under 365 nm-UVA, 302 nm-UVB, and 254 nm-UVC illumination (107.34 μW/cm²).

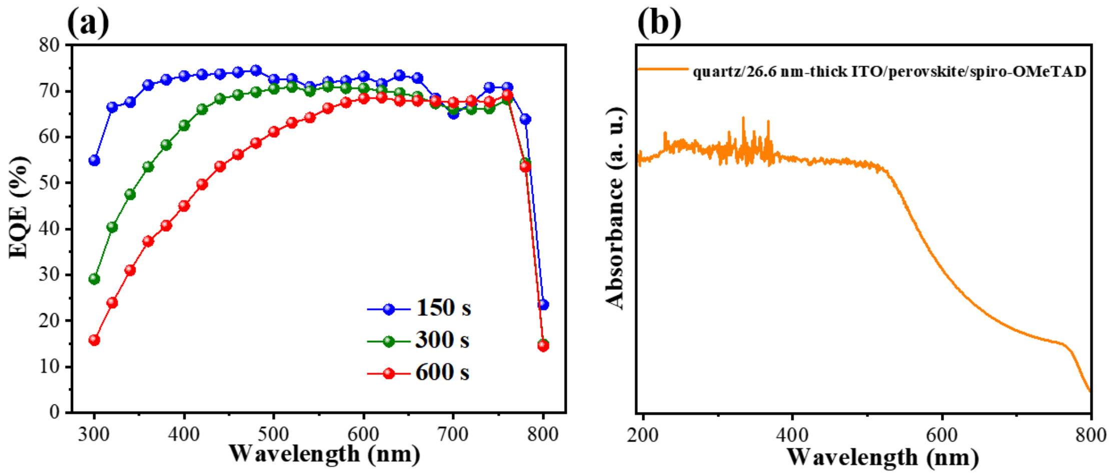

Figure S5. (a) The external quantum efficiency (EQE) plot as a function of wavelength, (b) UV-vis spectrum of the complete device.

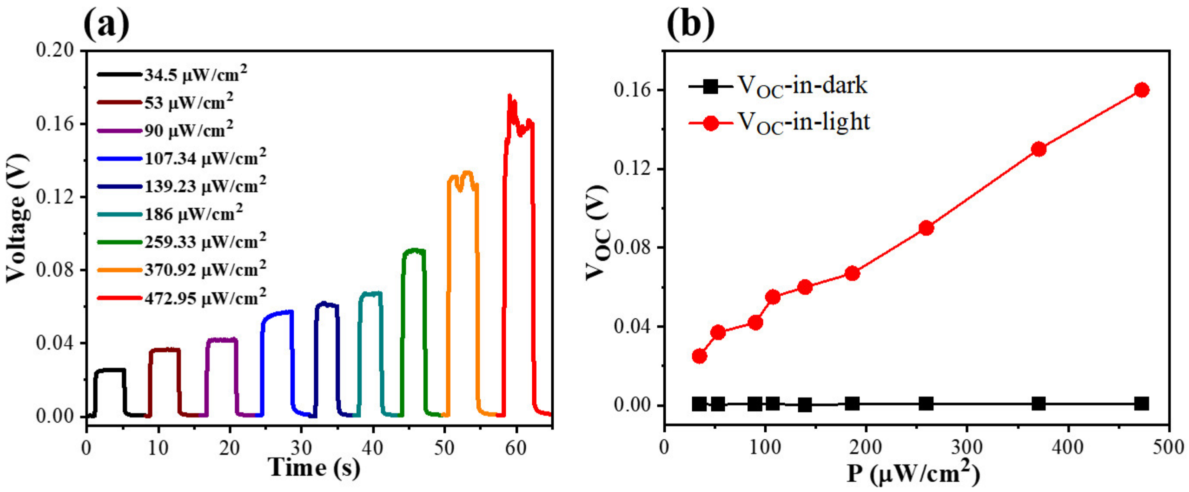

Figure S6. (a) The V-t plots in open-circuit mode of the prepared photodetector using 26 nm-thick ITO thin film under 254-nm UV illumination. (b) Photovoltage as a function of light intensity.
